# Bioengineered mesenchymal stem cell-derived exosomes: emerging strategies for diabetic wound healing

**DOI:** 10.1093/burnst/tkae030

**Published:** 2024-07-16

**Authors:** Lihua Liu, Dewu Liu

**Affiliations:** Medical Center of Burn Plastic and Wound Repair, The First Affiliated Hospital, Jiangxi Medical College, Nanchang University, Yongwaizheng Road, Donghu District, Nanchang, Jiangxi, P.R. China; Huankui Academy, Nanchang University, Xuefu Road, Honggutan District, Nanchang, Jiangxi, 330006, P.R. China; Medical Center of Burn Plastic and Wound Repair, The First Affiliated Hospital, Jiangxi Medical College, Nanchang University, Yongwaizheng Road, Donghu District, Nanchang, Jiangxi, P.R. China

**Keywords:** Mesenchymal stem cells, Exosomes, Diabetic wound healing, Bioengineering strategies, Application, Biomaterial

## Abstract

Diabetic wounds are among the most common complications of diabetes mellitus and their healing process can be delayed due to persistent inflammatory reactions, bacterial infections, damaged vascularization and impaired cell proliferation, which casts a blight on patients’health and quality of life. Therefore, new strategies to accelerate diabetic wound healing are being positively explored. Exosomes derived from mesenchymal stem cells (MSC-Exos) can inherit the therapeutic and reparative abilities of stem cells and play a crucial role in diabetic wound healing. However, poor targeting, low concentrations of therapeutic molecules, easy removal from wounds and limited yield of MSC-Exos are challenging for clinical applications. Bioengineering techniques have recently gained attention for their ability to enhance the efficacy and yield of MSC-Exos. In this review, we summarise the role of MSC-Exos in diabetic wound healing and focus on three bioengineering strategies, namely, parental MSC-Exos engineering, direct MSC-Exos engineering and MSC-Exos combined with biomaterials. Furthermore, the application of bioengineered MSC-Exos in diabetic wound healing is reviewed. Finally, we discuss the future prospects of bioengineered MSC-Exos, providing new insights into the exploration of therapeutic strategies.

Highlights

- This article reviews three bioengineering strategies, including parental MSC-Exos engineering, direct MSC-Exos engineering and MSC-Exos combined with biomaterials, to address challenges concerning the efficacy and yield of MSC-Exos.
- The application of bioengineered MSC-Exos in diabetic wound healing is reviewed.
- Bioengineered MSC-Exos provide new therapeutic strategies for diabetic wound healing.

## Background

Diabetes has become an increasingly prevalent chronic metabolic disease worldwide, with the number of individuals affected expected to increase to 643 million by 2030 and further to 783 million by 2045 [1]. The negative effects of diabetes are primarily manifested in its complications, particularly in the challenging healing process of diabetic wounds, which can result in lower limb amputation, not only negatively impacting and causing pain in the lives of patients but also increasing the demand for social health resources [2]. Hence, it is imperative to develop innovative and efficient techniques for expediting the healing of wounds in diabetic patients. Given the limited efficacy of traditional treatments such as debridement and wound dressings, novel approaches for diabetic wound treatment, including bioengineered skin substitutes, hyperbaric oxygen therapy, electrically stimulated pulsed therapy and stem cell therapy, have emerged [3]. Among them, stem cells have garnered increasing interest for their capacity for multidirectional differentiation, self-renewal and regeneration-promoting cytokine secretion. Exosomes derived from mesenchymal stem cells (MSC-Exos) have been demonstrated to play a therapeutic role in diabetic wound healing [4]. In contrast to stem cell therapy, therapy based on MSC-Exos eliminates the potential dangers of immune rejection and tumorigenesis associated with stem cell transplantation [5]. Therefore, the application of MSC-Exos therapy holds great potential in the treatment of wounds exacerbated by diabetes.

Although exosomes have distinct therapeutic advantages in managing diabetic injuries and the field is expanding rapidly, there are numerous obstacles that need to be addressed for the effective utilization of exosomes. (1) One major challenge is the absence of standardized preparation techniques and associated technical concerns, which can greatly impact the isolation, purification and large-scale manufacturing of exosomes, thereby impeding their clinical implementation [6]. (2) Exosomes may be rapidly cleared in the circulation or poorly retained in wounds [6]. Therefore, the incorporation of bioengineering methods into therapies based on MSC-Exos is anticipated to tackle the aforementioned practical applications. Bioengineered MSC-Exos possess specific genetic/conjugated surface proteins and bioactives that enable efficient interactions with target cells and targeted administration to specific locations. This maintains the superior biocompatibility, stability and low immunogenicity of exosomes while also enhancing therapeutic effectiveness, ultimately leading to improved optimization of the therapeutic efficacy of exosomes [7,8].

In this review, we primarily focus on bioengineering strategies and applications to enhance the effectiveness of MSC-Exos in diabetic wound healing. The functions and mechanisms of natural MSC-Exos in diabetic wound healing are presented and the obstacles they encounter are also summarized. Following that, the discussion revolves around bioengineering techniques aimed at improving the efficacy and yield of MSC-Exos, along with their advantages and challenges, with a focus on summarizing the application of bioengineered MSC-Exos in treating diabetic wounds. Finally, we outline the potential future of bioengineered MSC-Exos in the management of diabetic wounds.

## Review

### Normal and diabetic wound healing 

#### Normal wound healing

**Figure 1:**
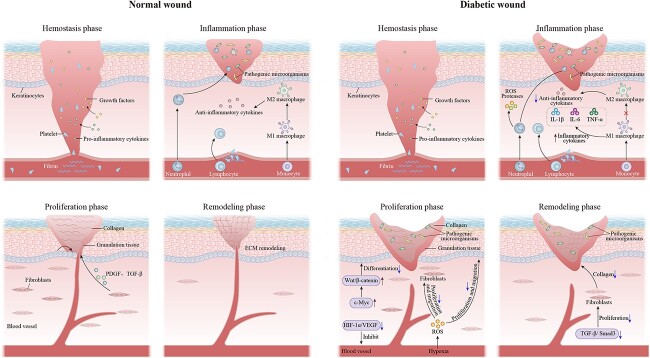
Comparison of the process between normal wound healing and a diabetic non-healing wound. Normal wound healing progress can be simplified into four main phases: hemostasis, inflammation, proliferation and remodeling. However, diabetic wounds are difficult to heal because of excessive inflammation, reduced angiogenesis, impaired keratinocytes and fibroblasts and lessened collagen deposition. The signals involved in diabetic wound pathology are elucidated. *ROS* reactive oxygen species, *IL-1β* interleukin-1beta, *IL-6* interleukin-6, *TNF-α* tumor necrosis factor alpha, *PDGF* platelet-derived growth factor, *TGF-β* transforming growth factor beta, *HIF-1α* hypoxia-inducible factor-1α, *VEGF* vascular endothelial growth factor, *ECM* extracellular matrix

The process of wound healing is intricate and ever-changing, necessitating the careful synchronization of multiple aspects, and has been simplified into four primary phases: hemostasis, inflammation, proliferation and remodeling [9] (Figure 1).

Hemostasis begins immediately after an injury by causing the blood vessels to narrow and form a clot made of fibrin [10]. Inflammatory cells and fibroblasts are induced to proliferate and migrate due to the release of proinflammatory cytokines and growth factors, such as platelet-derived growth factor and fibroblast growth factor (FGF), from the clot and the tissue surrounding the wound [11]. Subsequently, there is an inflammatory stage marked by persistent infiltration of neutrophils, macrophages and lymphocytes [12]. In the early phase of inflammation, neutrophils quickly move to the injured site to eliminate harmful microorganisms and then signal for the arrival of macrophages [13]. In the later stages of inflammation, macrophages undergo a shift in phenotype from M1 (proinflammatory) to M2 (anti-inflammatory) [14]. M2 macrophages secrete substances that have an anti-inflammatory effect and stimulate keratinocytes, fibroblasts and angiogenesis, ultimately supporting tissue regeneration [15]. Angiogenesis, granulation tissue formation, collagen deposition and epithelial formation are the primary processes involved in wound healing during the proliferative phase [9]. Fibroblasts and myofibroblasts in the surrounding tissues are stimulated to proliferate and subsequently migrate toward the injury site with the help of growth factors like platelet-derived growth factor and transforming growth factor-β (TGF-β) to generate collagen types I and III. The extracellular matrix (ECM) is formed as a result of this process, which is accompanied by the appearance of fresh capillaries and inflammatory cells, ultimately contributing to the formation of granulation tissue [16]. Furthermore, fibroblasts release growth factors that induce the proliferation and migration of keratinocytes, facilitating re-epithelialization of the wound and reconstruction of the epidermal barrier of the skin. During the remodeling phase, the ECM undergoes transformation to a structure similar to that of healthy tissue. Specifically, matrix metalloproteinases (MMPs) degrade the former ECM, causing the conversion of collagen III into collagen I, which decreases the size of the scar and improves its tensile strength [17].

### Pathological conditions in diabetic wound healing

In the context of diabetes, wound healing also proceeds through the above-mentioned steps. Nevertheless, numerous factors hinder the healing of diabetic wounds, including hyperglycemia, chronic inflammation, microcirculation, macrocirculatory dysfunction, hypoxia, and autonomic and sensory neuropathy [18].These factors impact the healing process by influencing the four stages and multiple pathways.

Hyperglycemia induces an imbalance in the polarization of macrophages and leads to increases in inflammatory cytokines such as interleukin-1β (IL-1β), IL-6 and tumor necrosis factor alpha (TNF-α), preventing the transition from M1 to M2 macrophages [19]. Chronic inflammation is caused by the prevalence of proinflammatory M1 macrophages in diabetic wounds and the scarcity or absence of anti-inflammatory M2 macrophages [20]. Moreover, neutrophils generate substantial quantities of reactive oxygen species (ROS) and proteases that destroy normal tissues. Alterations in the metabolism and function of endothelial cells result in various dysfunctions in both micro- and macro-circulation, ultimately impacting the process of angiogenesis [21]. In addition, despite the decrease in oxygen levels, diminished expression levels of hypoxia-inducible factor-1α (HIF-1α) and its target genes [e.g. vascular endothelial growth factor (VEGF)] in diabetic wounds contribute to an impaired cellular response to hypoxia. This notably affects angiogenesis, leading to delayed wound healing [22]. Insufficient blood supply causes hypoxia, resulting in oxidative stress and elevated levels of ROS, impairing the growth and movement of fibroblasts and keratinocytes, with a subsequent decrease in collagen deposition [23,24]. Keratinocytes are regulated by c-Myc, a significant protein involved in controlling epithelial–mesenchymal transformation. Nevertheless, c-Myc is overexpressed in the keratinocytes of diabetic wounds, resulting in the activation of the Wnt/β-catenin signaling pathway and subsequently causing impaired keratinocyte differentiation [25]. Additionally, the TGF-β/Smad3 pathway is suppressed in fibroblasts, leading to a decrease in collagen deposition due to hyperglycemic conditions [26]. Moreover, impaired nerve function in the feet of individuals with diabetes results in a decreased ability to feel pain from injuries, which hinders the process of wound healing [27]. Importantly, diabetic patients are susceptible to infections, further exacerbating the difficulty involved in healing diabetic wounds [28]. The various and intricate pathological states of diabetic wounds lead to a suboptimal environment for healing, highlighting the significance of addressing these factors for effective treatment.

### Overview of exosomes

#### Biogenesis of exosomes

**Figure 2:**
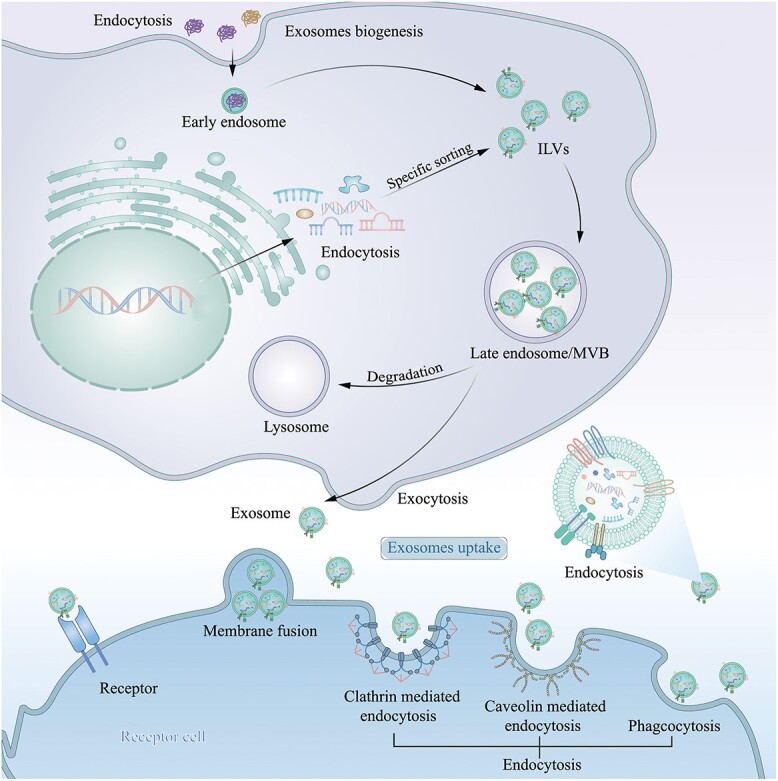
Exosome formation as a result of the inward budding of MVBs through endocytosis and release to the extracellular environment through exocytosis. The exosomes are taken up by recipient cells through three mechanisms: (1) receptor–ligand interaction, (2) direct fusion with the plasma membrane and (3) internalization. *ILVs* intraluminal vesicles, *MVB* multivesicular body

Exosomes originate from the endocytosis pathway and are released by multivesicular bodies (MVBs) [29]. Their biogenesis involves four stages: emergence, invagination, formation of MVBs and secretion [30]. First, the cell membrane undergoes invagination to form vesicles coated with lattice proteins, and then the lattice protein vesicles enter the cytoplasm to establish early intranuclear bodies. Proteins, lipids and nucleic acids are selectively packaged and meticulously enclosed to create ample intraluminal vesicles, which become precursors of exosomes [31]. Following the completion of late endocytosis, several intraluminal vesicles undergo subsequent growth and maturation, ultimately forming late endosomes/MVBs. A portion of the MVBs then follows the lysosomal pathway, whereas the rest fuse with the cell membrane and are ultimately excreted into the extracellular environment as exosomes [32] (Figure 2).

#### Biological function of exosomes

Exosomes are small vesicles enclosed by lipid bilayers, typically measuring between 40 and 150 nm in diameter. They are present in various body fluids such as urine, plasma, cerebrospinal fluid, breast milk and saliva. Additionally, they are released by different types of mammalian cells (e.g. MSCs, immune cells, cancer cells and neurons) [33]. Exosomes contain a variety of biologically active molecules, including proteins, messenger RNAs (mRNAs), transfer RNAs, and noncoding RNAs (ncRNAs) such as long noncoding RNAs and microRNAs (miRNAs). Due to their distinctive lipid bilayer structure, exosomes safeguard their cargos from degradation and further mediate intercellular communication through the transport of biologically active substances such as DNA, ncRNAs and more transferred from parental cells to recipient cells [34]. It has been demonstrated that MSC-Exos have inherited the therapeutic potential of stem cells contributing to diabetic wound healing, and participate in tissue repair and regeneration by exerting a crucial role in mediating intercellular signaling and effectively regulating immune responses, cell proliferation, migration and blood vessel production [35].

#### Uptake of exosomes in recipient cells

Upon reaching the target cell, exosomes can be taken up by the recipient cell through receptor–ligand interactions, direct membrane fusion or internalization. In receptor–ligand interactions, transmembrane proteins located on the surface of exosomes directly attach to the proteasome present on the target cell membrane, and consequently, the contents of exosomes selectively enter the target cell via permeable intracellular signals [36]. Membrane fusion then occurs between the recipient cell membrane and the exosome membrane, releasing the luminal cargos of exosomes into the cytoplasm of the recipient cell [37]. Exosomes can be internalized through various pathways during internalization, including lattice protein-mediated endocytosis, vesicle protein-mediated endocytosis, phagocytosis and macropinocytosis [37,38]. While nonspecific uptake pertains to all cell types, it is equally essential to specifically target receptor cells. The targeting action is facilitated by effective surface receptors for exosomes, intricate lipids and chemotaxis between exosomes and recipient cells [38]. This natural mechanism has inspired the design of bioengineered exosomes for targeting diabetic wound sites to exert vital therapeutic effects.

### Role of natural MSC-Exos in diabetic wounds

**Figure 3:**
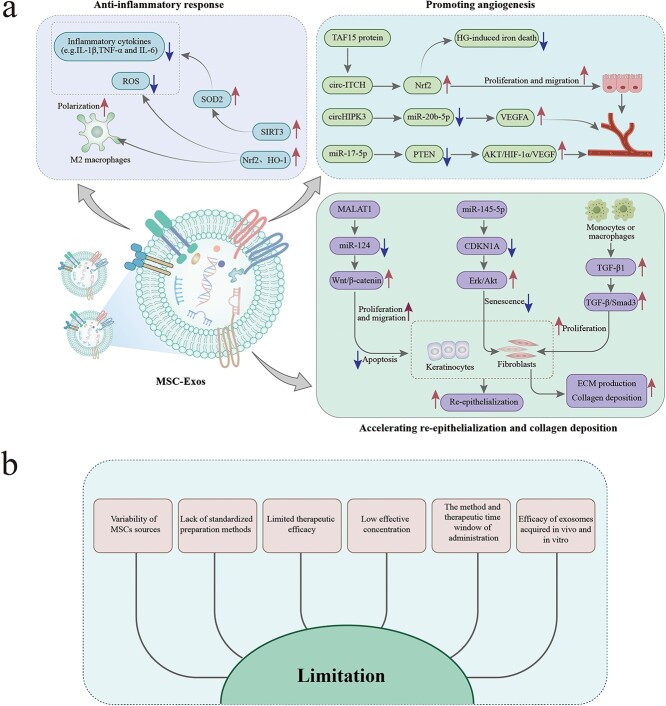
The overview of natural MSC-Exos based therapies in diabetic wounds (**a**) The role of MSC-Exos in diabetic wound healing. MSC-Exos can accelerate diabetic wound healing by inhibiting inflammation, promoting angiogenesis, as well as accelerating re-epithelialization and collagen deposition. (**b**) The limitation of the development of MSC-Exos-based therapies. Despite the therapeutic potential of MSC-Exos in clinical settings, they still face challenges in the development of MSC-Exos-based therapies. *MSC* mesenchymal stem cells, *Exos* exosomes, *Nrf2* nuclear factor E2-related factor 2, *HO-1* heme oxygenase-1, *SIRT3* Sirtuin 3, *SOD2* superoxide dismutase 2, *ROS* reactive oxygen species, IL-1β interleukin-1 beta, *TNF-α* tumor necrosis factor alpha, *IL-6* interleukin-6, *PTEN* phosphatase and tensin homolog, *AKT* protein kinase B, *HIF-1α* hypoxia-inducible factor-1α, *VEGF* vascular endothelial growth factor,* circHIPK3* circular RNA homeodomain-interacting protein kinase 3, *VEGFA* vascular endothelial growth factor-A,* circ-ITCH* circRNA-itchy E3 ubiquitin protein ligase, *TAF15* TATA-Box-binding protein associated factor 15, *HG* high glucose, *MALAT1* metastasis-associated lung adenocarcinoma transcript-1, *CDKN1A* cyclin-dependent kinase inhibitor 1A, *Erk* extracellular signal regulated kinase, *TGF-β1* transforming growth factor beta1, *ECM* extracellular matrix

MSCs, a class of adult stem cells that are capable of regenerating and differentiating in multiple ways, can be extracted from a variety of tissues, including umbilical cord, bone marrow, adipose tissue, skeletal muscle tissue and periodontal ligament [39]. Therefore, as a new approach for treating wounds in diabetic patients, the transplantation of MSCs has garnered significant interest [40]. However, cell therapies based on MSCs are associated with immune rejection as well as safety concerns [41]. Considering that the major therapeutic properties of MSCs depend on their paracrine functions, MSC-Exos may present a promising cell-free therapy to serve as a substitute for diabetic wound healing. Compared to MSCs, MSC-Exos are able to penetrate biological barriers and possess lower immunogenicity and tumorigenicity, avoiding the hidden risks of direct cell transplantation to a significant extent [42]. They can serve as ideal nanocarriers for therapeutic agents such as miRNAs, small interfering RNAs (siRNAs) and drugs, and their efficacy and targeting ability can be modified through bioengineering [5], illustrating promising prospects for the treatment of diabetic wounds. Research has indicated that natural MSC-Exos deliver bioactive molecules (e.g. growth factors, cytokines, ncRNAs and proteins) from MSCs to target cells, which can accelerate wound healing by inhibiting inflammation, promoting neovascularization, and stimulating collagen deposition and re-epithelialization [43–46] (Figure 3).

#### Anti-inflammatory response

Studies have demonstrated that MSC-Exos facilitate the polarization of M2 macrophages, reduce the expression of proinflammatory factors and decrease oxidative stress levels, thereby attenuating the chronic inflammation responsible for delayed healing in diabetic wounds. Nuclear factor E2-related factor 2 (Nrf2) is a significant transcription factor that regulates several cytoprotective genes, including heme oxygenase-1 (HO-1). HO-1 diminishes ROS generation and additionally enhances the transformation of macrophages to an anti-inflammatory phenotype to play a protective role [47]. Exosomes from adipose-derived MSCs (ADSC-Exos) can polarize macrophages toward the M2 anti-inflammatory phenotype by increasing Nrf2 and HO-1 levels, and alleviate the accumulation of LPS-induced ROS and inflammatory cytokines such as IL-1β, TNF-α and IL-6 in macrophages to prevent excessive inflammation [48]. In addition, a high-glucose (HG) environment can suppress sirtuin 3 (SIRT3), increase ROS, decrease superoxide dismutase 2 activity and elevate inflammatory cytokine levels. Zhang *et al*. [49] found that ADSC-Exos could decrease the expression of inflammatory cytokines to promote diabetic wound healing (e.g.VCAM-1, IL-1, IL-6 and TNF-α) by reinforcing the expression of SIRT3, boosting the activity of superoxide dismutase 2 and reducing the build-up of ROS.

#### Promoting angiogenesis

In addition to activating microvascular endothelial cells, MSC-Exos accelerate angiogenesis by promoting high levels of angiogenesis-related factors. circRNA-itchy E3 ubiquitin protein ligase from bone marrow MSC-Exos (BMSC-Exos) can inhibit ferroptosis and enhance angiogenesis in human umbilical vein endothelial cells (HUVECs) by activating the Nrf2 signaling pathway through the recruitment of the TAF15 protein to accelerate diabetic wound healing [50]. During the initial phases of angiogenesis, VEGF, a powerful proangiogenic factor, promotes the growth and migration of endothelial cells. Liang *et al*. [43] demonstrated that circular RNA homeodomain-interacting protein kinase 3 contained in exosomes from human umbilical cord MSCs (HUCMSC-Exos) might expedite diabetic wound healing by functioning as a competitive endogenous RNA (ceRNA) inhibitor of miR-20b-5p to suppress its interaction with the downstream gene VEGFA and thereby lead to the enhancement of VEGFA expression, consequently facilitating cell proliferation, migration and angiogenesis. Wei *et al*. discovered that extracellular vesicles from HUCMSCs exerted a protective and vigorous effect on endothelial cells exposed to HG through miR-17-5p by suppressing phosphatase and tensin homolog (PTEN) expression and stimulating the protein kinase B (AKT)/HIF-1α/VEGF pathway, ultimately expediting diabetic wound healing [51].

#### Accelerating re-epithelialization and collagen deposition

Numerous studies have indicated that MSC-Exos can modulate the proliferation and migration of fibroblasts and keratinocytes, expedite the process of re-epithelialization, and influence collagen deposition by regulating related genes and growth factors. Metastasis-associated lung adenocarcinoma transcript-1-containing ADSC-Exos inhibit apoptosis and enhance the proliferation and migration of H_2_O_2_-injured fibroblasts and keratinocytes by interacting with miR-124 and activating the Wnt/β-catenin pathway, thereby facilitating re-epithelialization [46]. HG-induced senescence of fibroblasts in diabetic wounds leads to insufficient ECM production and collagen deposition [52]. A recent study revealed that exosomes from placental MSCs boosted the function of HG-induced senescent fibroblasts via miR-145-5p targeting cyclin-dependent kinase inhibitor 1A to activate the extracellular signal regulated kinase/Akt signaling pathway, contributing to accelerating diabetic wound healing [53]. Among the complex signaling pathways engaged in collagen synthesis, the TGF-β/Smad3 pathway performs a vital role. It was found that ADSC-Exos can enhance the secretion of TGF-β1 by monocytes or macrophages, resulting in the stimulation of fibroblast proliferation through activation of the TGF-β/Smad3 signaling pathway, resulting in increased production of collagen I in diabetic wounds [44].

### Challenges in the development of MSC-Exos-based therapies

As previously stated, MSC-Exos can promote diabetic wound healing, regulate cellular behavior and function by transporting genetic material and transcription factors, modulate the inflammatory and immune microenvironment, and promote angiogenesis and collagen deposition through cell–cell interactions [5]. Notably, despite the considerable potential offered by MSC-Exos in clinical settings, they still face challenges. First, unresolved issues include the variability in the origin of parental MSCs, the procedures for isolating, purifying and identifying of exosomes, and the establishment of standardized definitions for MSC-Exos [54]. Second, certain RNAs or proteins in MSC-Exos may have limited quantities or inadequate targeting capabilities, rendering them ineffective for wound treatment [55]. Furthermore, the effectiveness of MSC-Exos in therapy might be impacted by the surrounding microenvironment, the method of administration and the therapeutic time window of intervention [54]. In addition, the permeability, accumulation and retention of exosomes in wounds are typically restricted by the rapid clearance of the drug administered systemically, caused by blood circulation, as well as by the movement of body fluids when exosomes are administered externally [56]. More importantly, we also have to take into account the potential variations in therapeutic outcomes when comparing exosomes obtained from MSCs cultured *in vitro* vs. exosomes secreted by MSCs following *in vivo* administration. Hence, researchers are actively investigating new bioengineering strategies to improve the therapeutic potential of MSC-Exos in diabetic wound repair.

**Figure 4:**
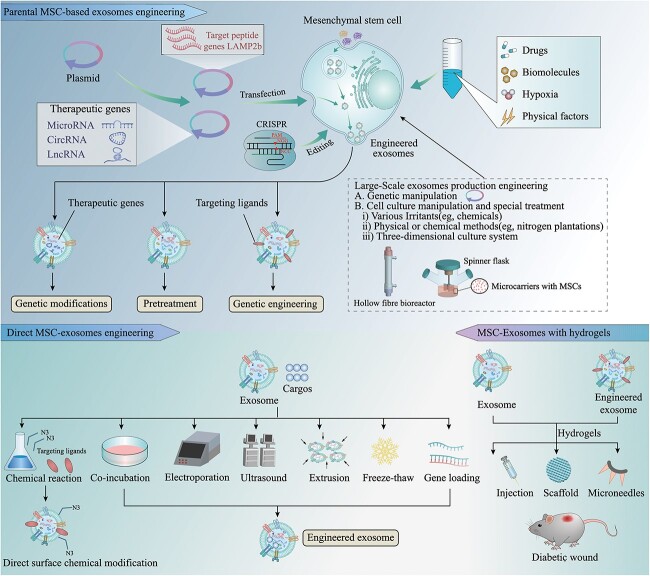
Modification strategies for bioengineered MSC-Exos, including engineering of parental MSCs, direct MSC-Exos engineering and MSCs-Exos combined with biomaterials. *MSCs* mesenchymal stem cells, *Exos* exosomes, *CircRNA* circular RNA, *LncRNA* long noncoding RNA, *CRISPR* clustered regularly interspaced short palindromic repeats

### Bioengineering improves the efficacy and yield of MSC-Exos

To augment the therapeutic role of exosomes in diabetic wound healing, the low effective concentration of MSC-Exos and their limited therapeutic efficacy need to be addressed. Researchers have introduced various bioengineering techniques into MSC-Exos to improve their efficacy and yield. Under the prevailing circumstances, bioengineering strategies are categorized into three main groups: parental MSC-Exos engineering, direct MSC-Exos engineering and MSC-Exos combined with biomaterials (Figure 4).

#### Parental MSC-Exos engineering

Modification of parental MSCs prior to obtaining exosomes is one way to obtain bioengineered MSC-Exos. Parental cells can be genetically engineered with modifications to enhance the cellular targeting ability of exosomes. In addition, parental MSCs can be genetically modified or pretreated to enhance exosome function. Moreover, engineering strategies have been utilized to enhance exosome yield for practical applications.

##### Genetic engineering

Despite the potential, there are still certain limitations to the use of natural exosomes as delivery platforms. For example, exosomes not only reach the injury location, but distribute the payload to all cell types in the body indiscriminately, thereby lessening the availability of miRNAs. Consequently, genetic engineering methods modify the transmembrane structural domains of exosomes to achieve cell-specific targeting [57].

Genetic engineering uses gene transfer vectors (plasmid/virus) to achieve the expression of genes for targeted parts (e.g. peptides, receptors and antibodies) in parental cells by transfection/transduction to produce exosomes with targeted peptides on their surfaces during the process of natural exosome biogenesis [58]. The most common application of loading targeted peptides onto exosome surfaces by genetic engineering is to utilize exosome signaling peptides such as lysosome-associated membrane protein 2b (LAMP2b) [59], as peptides have the advantages of being small, having high binding affinity, being highly targeted and being designed on the basis of the innate mechanism of the natural ligand–receptor interaction of exosomes [60]. Genetic modification of cell-specific binding peptides at the N-terminus of LAMP2b [61] could be used to target specific organs or tissues. As described by Yang *et al*. [62], BMSCs were transfected with the pcDNA3.1(−)-rabies virus glycoprotein-Lamp2b plasmid, which enabled the presentation of rabies virus glycoprotein on the surface of exosomes. This modification allowed effective targeting of miR-124 delivery to the infarct site. Further studies revealed that exosomes containing chondrocyte affinity peptide (DWRVIIPPRPSA), designed by the researchers to specifically transport miRNA-140 directly to chondrocytes in the joints, mitigated the advancement of osteoarthritis in rats [63]. Moreover, other exosome surface proteins including tetraspanins (CD63, CD9 and CD81) [64], T-calmodulin (a unique glycosylphosphatidylinositol-anchored calmodulin) [65], platelet-derived growth factor receptors [66] and lactoadhesins (C1C2 structural domains) [67], can also serve the same function. In addition, exosomes have the ability to attach to ECM constituents in a manner that resembles the sequestration of growth factors in the ECM [68]. The presence of adhesion receptors on exosome membranes could account for the interaction between exosomes and the ECM, suggesting that the integration of instructive biomolecule signals into exosome membranes can improve the binding efficiency of exosomes to biomaterials. A study conducted recently involved the construction and transduction of silk fibroin-binding peptide-Gluc-MS2 into human placenta-derived MSCs. During exosome formation, incorporating silk fibroin-binding peptide into the exosomal membrane ultimately led to increased affinity of exosomes for silk fibroin patches [69].

The surface display of peptides and proteins is effectively achieved through the implementation of this exosomes targeting strategy. Nevertheless, its scope is restricted to focusing on genetically codable patterns [61]. Additionally, this strategy is constrained by the stringent isolation techniques necessary for producing engineered exosomes with high purity.

##### Genetic modification

Selected parental MSCs can be genetically modified by transfection or transduction using plasmids or lentiviruses carrying the target cargos to load therapeutic nucleotides or proteins that result in increased or silenced expression of exosome genes, increasing their efficacy. For example, Yuan *et al*. [70] transfected ADSCs to establish miR-29-overexpressing cells, and exosomes derived from miR-29a-modified ADSCs reduced excessive scar formation by blocking the TGF-β2/Smad3 pathway. Furthermore, gene editing of parental MSCs can also be performed using clustered regularly interspaced short palindromic repeats (CRISPR)/Cas9 technology to modify the abundance of miRNAs in exosomes. For instance, the utilization of CRISPR/Cas9 has resulted in the knockout of specific gene sequences in UMSCs, yielding exosomes that exhibit greater efficiency than those obtained from UMSCs [71]. Although CRISPR/Cas9 technology has not yet been widely applied to MSC-Exos-based therapy for diabetic trauma, the advantages that make site-specific gene targeting possible give it great potential for application [72]. Moreover, magnetic nanoparticles, like iron oxide nanoparticles, have garnered attention in gene delivery due to their low immunogenicity, high efficiency, non-invasiveness and cost-effectiveness [73]. Magnetic nanoparticle transfection involves utilizing these nanoparticles as carriers to deliver nucleic acid molecules (e.g. exogenous DNA or RNA) into target cells under a magnetic field, which enables targeted transfection of cells and optimizes transfection efficiency [74]. Consequently, magnetic nanoparticle transfection holds significant promise for therapeutic gene delivery in tissue engineering repair. A recent study [75] revealed that under the co-stimulation of an electromagnetic field and polyethyleneimine-coated iron oxide nanoparticles, miR-21 was successfully introduced into BMSCs and HUVECs via the magnetic transfection system, markedly boosting transfection efficiency championed by the p38 MAPK pathway activated by the magnetic transfection system, and, in combination with polycaprolactone and hydroxyapatite scaffolds, contributing to osteogenesis and angiogenesis in lumbar degenerative disc disease. Since magnetic nanoparticle transfection has demonstrated tremendous therapeutic potential for gene modification in MSCs, the prospect for progressive research appears bright despite the scarcity of robust research evidence for its application in MSC-Exos currently.

##### Pretreatment of parental MSCs

To enhance the biological functions as well as the efficacy of exosomes, drugs, biomolecules, hypoxic conditions and physical factors can be applied to the culture environment of parental MSCs before treatment. For example, MSCs can be treated with fluoxetine [76], dimethyloxaloylglycine [77], salidroside [78] and hypoxia [79] to augment their paracrine or regenerative capabilities. Furthermore, released exosomes are capable of inheriting biological properties from their pretreated parental MSCs and achieving therapeutic effects through bioactive molecules in the lumen. It was shown that pretreatment of HUCMSCs with 3,3′-diindolylmethane activated exosomal Wnt11 autocrine signaling and promoted dryness of HUCMSCs, accelerating deep second-degree burn wound healing [80]. Besides, exosomes activated by selenium improve the healing by modulating inflammation and angiogenesis [81]. Despite the inability of pretreatment to directly alter particular cargos, exosomes retain certain bioactive molecules or biological characteristics from their origin cells, benefiting the therapeutic results accordingly.

##### Mass production exosome engineering

Despite the promising future of MSC-Exos in diabetic wound therapy, their application is generally restricted due to their low yields under conventional culture conditions. Typically, two approaches have been employed to enhance exosome manufacturing. One strategy is genetic manipulation, which involves overexpressing activation genes engaged in exosome formation and downregulating relevant genes in the exosomes recycling pathway [82]. For instance, with the guide of genetic manipulation, exosome secretion can be significantly enhanced by biogenesis activation (such as the overexpression of heat shock protein and tetraspanin) and inhibition of exosome recycling (such as phosphocreatine kinase and negative regulation of FYVE-type zinc fingers) [83]. Another strategy is cell culture manipulation and specific drug treatments. MSCs are stimulated to produce more natural exosomes by various stimuli in the environment, such as chemicals, protein regulation, heat and oxidative stress, oxygen levels, pH, radiation, starvation and culture methods. However, the majority of these stimulation methods impose stress on cells and interfere with the cellular contents of exosomes. Additionally, physical and chemical techniques such as nitrogen cavitation, nitrogen implantation, sonication, extrusion through porous membranes, disruption of cell membranes by high pH solubilization, and reconfiguration to release contents can be used to synthesize artificial exosomes [84]. When utilized as therapeutic agents, there is an increased likelihood of eliciting an immune response within living organisms compared to natural exosomes [85]. Furthermore, employing a 3D culture platform could prove to be a viable approach for enhancing exosome production in the clinic. At present, bioreactors such as hollow-fiber bioreactors [86] and microcarriers [87] are utilized for cultivating cells on a large scale in a 3D environment. 3D spherical culture has been reported to induce therapeutic effects of MSCs, including reducing inflammation and promoting blood vessel growth, in addition to improving exosome production [88]. Moreover, biomaterials like 3D-printed scaffolds are engaged in cell culture and influence exosome yield and biological activity by mimicking the optimal physiological conditions for cell growth and exosome release within a dynamic 3D setting [89]. Interestingly, cellular nanoporation (CNP) has succeeded as an innovative technology for mass production of functional exosomes. Yang *et al*. [90] developed CNP biochips to stimulate cells to generate and release abundant exosomes containing therapeutic mRNAs and targeted peptides, which addressed the issue of low yield of functionalized exosomes and simplified the process of endogenous mRNA transcription in exosomes. CNP produced a 50-fold increase in exosomes numbers and a >1000-fold increase in exosomal mRNA transcripts compared to traditional bulk electroporation and other exosome-production strategies. Functionalized exosomes obtained based on the CNP technology were confirmed to have promising therapeutic effects in glioma mice [90] and photodamaged mice [91]. Increasing exosome production while maintaining or enhancing their efficacy by engineering the extracellular environment and intracellular components of MSCs remains a future endeavor.

#### Direct MSC-Exos engineering

Strategies for direct MSC-Exos engineering in recent years are summarized in this section. These strategies encompass the chemical modification of exosome surfaces for refined targeting, co-incubation of exosomes with cargos, electroporation and sonication, along with the rarely used freeze–thaw and extrusion methods. In these strategies, the loading efficiency depends on the hydrophilicity, hydrophobicity and molecular weight of the cargos as well as the stability and integrity of the exosome membrane [92].

##### Chemical modification of exosome surfaces

Chemical modifications of exosome surfaces enhancing targeting ability can be categorized into covalent or noncovalent action approaches. Covalent interaction is the addition of azide-containing compounds such as tetra-acetylated *N*-azidoacetyl-D-mannosamine to the surface of exosomes to create an active chemical site that allows azide-alkyne cycloaddition or click chemistry to couple orthogonally with the target peptide biologically without altering exosome size or function [93]. Tian *et al*. utilized click chemistry to specifically conjugate the cyclo peptide, which has a high affinity for integrin αvβ3 in reactive cerebral vascular endothelial cells after ischemia, onto MSC-Exos surfaces, which enhanced its ability to target the lesion region of the ischemic brain compared to that of unmodified exosomes [94]. Hydrophobic insertion is a commonly used noncovalent strategy for stabilizing modifications of biological membranes. The targeting portion is believed to be inserted directly into the exosome membrane because of the hydrophobic interactions caused by the lipid bilayer of exosomes [95]. For example, based on exosomes of induced MSCs (induced pluripotent stem cell derivatives, a new MSC subtype), Cui *et al*. anchored diacyllipid-modified bone-targeting peptides to exosomal membranes via hydrophobic insertion, resulting in significant enhancement of osteoblast targeting [96]. Furthermore, Wen *et al*. [97] first demonstrated that fucosyltransferase VII-mediated α-1,3-exofucosylation converted CD44 on the surface of hUCMSC-Exos into hematopoietic cell E-selectin/L-selectin ligand, significantly enhancing the tendency for mouse bone marrow homing compared to unmodified exosomes. This motivates us to attempt to pinpoint a molecule specifically expressed on macrophages, endothelial cells, fibroblasts or keratinocytes in diabetic wounds that would contribute to improving the specific targeting ability of engineered exosomes.

##### Co-incubation

Co-incubation is a commonly employed approach for preserving the integrity of exosome membranes, especially with regard to incorporating hydrophobic substances such as medications and RNAs. Through co-incubation, Guo *et al*. [98] efficiently loaded PTEN-siRNA into MSC-Exos without altering exosome size distribution or integrity, enhancing the repair of complete spinal cord injury in rats. However, as the lipid surface of exosomes comes into contact with the loaded hydrophobic molecules, the loading efficiency is significantly influenced by the hydrophobic nature and concentration gradient of the cargos [99]. Hence, it is challenging to control exosome loading due to the physicochemical nature of exosomes and the manner in which they are used for therapeutic purposes.

##### Electroporation

Electroporation is the most predominant passive strategy for loading cargos into exosomes, offering superior loading rates in comparison to incubation techniques. By applying an electric field, temporary pores are formed in the exosome membrane, allowing the drug to diffuse and enabling the integrity of the exosome membrane to be restored. To date, electroporation has been used to encapsulate a variety of cargos, including mRNAs, functional miRNAs and siRNAs, thereby reducing RNA degradation in the trauma microenvironment [100]. For example, hUCMSC-Exos modified with surface-engineering strategies could efficiently deliver miR-34c-5p via electroporation to selectively target leukemia stem cells, thereby impeding the development of acute myeloid leukemia [97]. Furthermore, Cui *et al*. analyzed thousands of miRNAs by conducting next-generation sequencing and demonstrated that loading siRNA of the Shn3 gene into MSC-Exos via electroporation had no effect on the endogenous miRNAs carried by exosomes. Correspondingly, the results indicated that the delivery of siRNA to osteoblasts facilitated the treatment of osteoporosis [96]. Nonetheless, it is still a requirement to oversee the procedure and optimize the volume ratio (exosomes/drug-containing medium), and exosomes need to be recharacterized after loading to ensure that their therapeutic effect is retained [101].

##### Sonication

Exosome engineering commonly utilizes sonication as a well-liked approach to enhance the administration of genes and medications. This approach utilizes homogenizer probes to shear the exosome membrane and allows exogenous cargos to diffuse into the exosomes through the newly created wells. Compared to co-incubation and electroporation, sonication exhibits greater loading efficiency; moreover, compared with co-incubation, it also effectively maintains exosome stability and facilitates cargo loading [102]. Nevertheless, high-power ultrasound may lead to permanent changes in exosomes, destroying the integrity of the membrane. Further studies used ultrasound to design a delivery technique capable of locally releasing exosomes at the desired location. Researchers intravenously injected exosomes together with a SonoVue™ microbubbles (Bracco Imaging) into mice and then disrupted these microbubbles by ultrasound targeting. The results showed that ultrasound-targeted microbubble disruption significantly increased exosome infiltration and endocytosis. However, the drawback is that exosomes are rapidly metabolized by the blood circulatory system, resulting in limits to their utilization [103].

##### Extrusion and freeze–thaw

Extrusion involves the introduction of a mixture of drugs and exosomes into an extruder so that exogenous cargos (e.g. gold nanoparticles) can be loaded into exosomes by disrupting the membrane [104,105]. The freeze–thaw method loads water-soluble molecules into exosomes by creating ice crystals within the lipid membrane, which causes temporary disruption of the exosome membrane [99]. According to previous studies, both extrusion and freeze–thaw techniques possess notable limitations, including reduced efficiency, diminished flow and the potential to compromise the stability of the exosome membrane [102,105]. As a result, both methods are rarely used to load cargo into exosomes.

#### MSC-Exos combined with biomaterials

Diabetic wound healing requires prolonged and recurring injections while free exosomes are rapidly removed. To tackle this issue, researchers have carried out multiple investigations on the combination of MSC-Exos with biomaterials to formulate slow-release therapeutic systems [106].

Numerous biomaterials, such as semipermeable membranes, semipermeable foams, electrospun nanofibers, colloidal nanoparticles, hydrocolloids and hydrogels, have been developed to facilitate wound repair. Among them, hydrogels have gained considerable interest as leading contenders for wound dressings because of their biocompatibility, good hydrophilicity and 3D porous structures such as ECM [107]. Hydrogels possess characteristics that render them appropriate for the encapsulation of exosomes, enabling gradual release at designated sites to provide a transient ECM for cellular infiltration and adhesion [108]. According to a comparative study, hydrogel–exosome complexes were found to significantly enhance diabetic wound healing in comparison to exosomes alone [109].

Over the last decade, extensive research has been conducted on hydrogel wound dressings. A wide range of hydrophilic polymers, including natural ones like chitosan [110] and silk fibroin [111], as well as synthetic ones such as polyethylene glycol (PEG) [112] and polyvinyl alcohol [113], have been used to construct hydrogels with various chemical or physical crosslinks in combination with MSC-Exos. Among them, natural polymers stand out due to their excellent biocompatibility and fostering of cell growth and differentiation. While natural hydrogels are commonly used in exosome delivery, they often require crosslinking with other polymers for enhanced stability, mechanical strength and tissue adhesion [114]. In contrast, synthetic hydrogels offer tailored properties like specific porosity, degradation time and mechanical strength, but lack the innate biological features of natural polymers [115]. Consequently, researchers are increasingly turning to hybrid hydrogels made from a combination of natural and synthetic materials to work together with MSC-Exos in the process of tissue regeneration and repair [116–118]. Furthermore, the function of hydrogels has evolved from being solely a physical covering or performing a single function to now encompassing a combination of multiple functions, indicating a growing inclination toward more intelligently designed offerings [119]. Given that the release of exosomes is not regulated by time, temperature or other stimuli, there is an urgent requirement for intelligent hydrogels that can respond to various stimuli (e.g. temperature, pH and enzymes) [120].

To summarize, despite the promising therapeutic potential of MSC-Exos demonstrated in recent preclinical studies, various limitations hinder their clinical application. Yield and efficacy issues need to be addressed when introducing exosomes into practical applications. The development of bioengineering methods based on MSC-Exos therapy could offer a fresh perspective.

### Bioengineered MSC-Exos in diabetic wound healing

The preceding discussion provides an overview of the bioengineering techniques available for modifying MSC-Exos. Compared with untreated MSC-Exos, bioengineered MSC-Exos possess notable attributes, including enhanced specificity, the capacity to carry drugs, proteins or genetic material within exosomes or on their exterior, as well as benefits like exceptional purity and abundant production. In this section, we review the applications of bioengineered MSC-Exos in diabetic wound healing at the level of three bioengineering technologies that better promote diabetic wound healing through anti-inflammatory effects, promotion of cell proliferation, and facilitation of angiogenesis and ECM remodeling (Table 1).

**Table 1:** Application of bioengineered MSC-Exos in diabetic wound healing

Engineering strategy		Exosome source	Nomenclature	Method	Function	Reference
Parental MSC-based exosomes engineering	Gene modification of parental MSCs	HBMSCs	EVs	HBMSCs transfected to overexpress long non-coding RNA HOTAIR	Promote angiogenesis and wound healing in diabetic mice	[121]
		ADSCs	Exos	RNA overexpression or interference was induced by transfection of circ-Astn1 to modify ADSCs	Enhance wound healing by promotion of angiogenesis and suppression of apoptosis through reducing miR-138-5p expression, promoting SIRT1 and suppressing FOXO1 expression in diabetic mice	[122]
		ADSCs	Exos	ADSCs transfected to overexpress mmu-circ-0000250	Promote wound healing in diabetes by absorption of miR-128-3p and upregulation of SIRT1 and suppressing apoptosis by autophagy activation	[123]
		ADSCs	Exos	ADSCs transducted to overexpress miR-132	Augment diabetic wound healing and skin reconstruction by reducing inflammation, enhancing angiogenesis and stimulating M2-macrophage polarization	[124]
		HMSCs	Exos	Parental MSCs co-transfected with plasmids encoding production enhancement genes	Increase exosomes production by 15- to 40-fold as determined by measuring a luminescent reporter	[125]
	Pretreatment of parental MSCs	HBMSCs	Exos	Melatonin preconditioned MSCs	Promote diabetic wound healing by suppressing the inflammatory response, which was achieved by increasing the ratio of M2 polarization to M1 polarization through activating the PTEN/AKT signaling pathway, thereby further facilitating angiogenesis and collagen synthesis *in vivo*.	[14]
		BMSCs	Exos	Atorvastatin preconditioned MSCs	Accelerate diabetic wound repair by promoting the proliferation, migration, tube formation and VEGF level of endothelial cells *in vitro* via the AKT/eNOS pathway by upregulating miR-221-3p	[126]
		HUCMSCs	Exos	Nr-CWS preconditioned HUCMSCs	Facilitate the proliferation, migration and tube formation of endothelial cells *in vitro* and advance diabetic wound healing by facilitating angiogenesis via the circIARS1/ miR-4782-5p/VEGFA axis *in vivo*	[127]
		ADSCs	Exos	HADSCs were subjected to hypoxia conditions (1% O_2_/5% CO_2_/94% N_2_) for 24 h	Accelerate skin wound healing in diabetic mice by regulating inflammatory factors and chemokines，promoting fibroblast proliferation and migration and extracellular matrix formation by PI3K/Akt pathways	[35]
		ADSCs	Exos	ADSCs were cultured in a hypoxic environment consisting of 5% CO_2_, 2% O_2_ and 93% N_2_ for 12 h	Enhance wound healing in diabetic mice via delivery of circ-Snhg11 and induction of M2-like macrophage polarization via the miR-144–3p/HIF-1α axis	[79]
Direct MSC-exosomes engineering	Exosome loading with genes	MSCs	Exos	MSC-derived exosomes loaded with miR-155 inhibitor by calcium chloride-modified method	Show synergistic effects in keratinocyte migration, restoration of FGF-7 levels and anti-inflammatory action, and enhance collagen deposition, angiogenesis and re-epithelialization in diabetic wounds	[129]
		ADSCs	Exos	miR-21-5p mimics were loaded into hADSC-Exos by electroporation	Promote proliferation and migration of keratinocytes via Wnt/β-catenin signaling *in vitro* and accelerate diabetic wound healing by increasing re-epithelialization, collagen remodeling, angiogenesis and vessel maturation *in vivo*	[55]
		MSCs	Exos	A known cell-penetrating peptide, YARA, was covalently conjugated with miR-21- 5p and loaded into MSC-Exos	Enhance the proliferation, migration and invasion of human and mouse fibroblasts thereby accelerating wound healing	[131]
Exosomes-incorporated delivery system	Stimuli-responsive hydrogels with Exos	ADSCs	Exos	An injectable, self-healing and antibacterial polypeptide-based FHE hydrogel possessing long-term pH-responsive bioactive exosomes release behavior was developed	Enhance diabetic full-thickness cutaneous wounds healing, characterized by enhanced wound closure rates, angiogenesis, re-epithelialization and collagen deposition	[120]
		ADSCs	Exos	Exos were loaded into the matrix metalloproteinase degradable polyethylene glycol (MMP-PEG) smart hydrogel	The smart hydrogel with enzyme-response and exosome-release was proved to promote diabetic wounds healing by optimizing cellular functions and relieving oxidative stress	[119]
		HUCMSCs	Exos	Exos combined pluronic F127 hydrogel with unique heat-sensitive properties	Compared with hUCMSC-Exos, the combination of PF-127 and Exos resulted in significantly accelerated wound closure rate	[109]
		MSCs	Exos	MSC-Exos-encapsulated adjustable poly(vinyl alcohol) (PVA) hydrogel served as microneedle tips	Promote wound healing in diabetic rat models by effectively activating fibroblasts, vascular endothelial cells and macrophages	[113]
	Hydrogels with improved properties	ADSCs	Exos	Exosome laden oxygen releasing antioxidant and antibacterial cryogel wound dressing OxOBand	Promote diabetic wounds by facilitating faster wound closure, enhancing collagen deposition, faster re-epithelialization and increasing neo-vascularization, and decreasing oxidative stress	[133]
		ADSCs	Exos	Exos are first loaded into Ag@ BSA nanoflowers to form a protective ‘pollen-flower’ delivery structure further encapsulated into the injectable collagen hydrogel	Remodel diabetic wound microenvironment for accelerating wound healing and promote blood perfusion, tissue granulation, collagen deposition,neovascularization, angiogenesis, and re-epithelialization	[134]
		ADSCs	Exos	A self-healing, tissue adhesive, antioxidant, provascular hydrogel loaded with 4-Arm-PEG-thiol, Ag, exosomes, CNTs and metformin hydrochloride was designed	Involve reducing the level of reactive oxygen species by interfering with mitochondrial fission, thereby protecting F-actin homeostasis and alleviating microvascular dysfunction to promote diabetic wound healing	[135]
		BMSCs	Exos	Extrusion-based cryogenic 3D printing technology constructed decellularized small intestinal submucosa combined with MBG and exosomes to fabricate a 3D scaffold dressing	Accelerate diabetic wound healing through stimulating the angiogenesis process and also promote granulation tissue formation, well-organized collagen fiber deposition	[136]
	Hydrogels with engineered Exos	ADSCs	Exos	Hypoxia-pretreated ADSC-Exos were embedded in GelMA hydrogels	Have a loose porous structure, and a stable degradation and expansion rate *i*n vitro and promote wound healing in diabetic mice *in vivo*	[137]
		HADSCs	Exos	Reductive 2D COFs as a nanocarrier to immobilize engineered exosomes collected from TNF-α-treated MSCs under hypoxia	Suppress oxidative injury and tissue inflammation, promote angiogenesis and eradicate bacterial infection to accelerate infected diabetic fester wound healing	[138]
		PMSCs	Exos	The engineered exosomes with enhanced targeting function were modified to efficiently load miR146a and attached to SFP	Promote diabetic wound healing by targeting IRAK1 associated with less inflammation, collagen deposition and neovascularization	[69]

*MSCs* mesenchymal stem cells, *Exo*: Exosomes, *HBMSCs* human bone marrow-derived mesenchymal stem cells, *EVs* extracellular vesicles, *HOTAIR* HOX transcript antisense RNA, *ADSCs* adipose-derived mesenchymal stem cells, *HUCMSCs* human umbilical cord mesenchymal stem cells, *VEGF* vascular endothelial growth factor, *Nr-CWS* Nocardia rubra cell wall skeleton, *MBG* mesoporous bioactive glass, *BSA* bovine serum albumin, *PMSCs* placental mesenchymal stem cells *SFP* silk fibroin patch, *IRAK1* interleukin-1 receptor-associated kinase 1

#### Parental MSC-Exos engineering in diabetic wound healing

First, genetic modification of parental MSCs is a convenient and reliable method. Enhancing diabetic wound healing is achieved by isolating exosomes that transport specific cargos from genetically modified donor cells. By utilizing a pCMV-HOTAIR plasmid, Born *et al*. [121] used BMSCs transfected with HOTAIR, a long noncoding RNA HOX transcript antisense RNA, to produce exosomes, which enhanced angiogenesis and wound healing in diabetic mice compared to natural MSC-Exos therapy. Furthermore, in contrast to wild-type ADSC-Exos, exosomes isolated from circ-Astn1-modified ADSCs that employed circDNMT3B adeno-associated virus markedly enhanced wound healing in diabetic mice through the regulation of the miR-138-5p/SIRT1/FOXO1 axis [122]. Equally, capitalizing on the circDNMT3B adeno-associated virus, it was further found that exosomes from mmu_circ_0000250-modified ADSCs exhibited a more potent curative effect on diabetic wound healing *in vivo* when compared to wild-type exosomes obtained from ADSCs [123]. Similarly, exosomes isolated from miR-132-overexpressing ADSCs supported by lentiviral transduction evidently augmented diabetic wound healing and skin reconstruction in mice by reducing inflammation, enhancing angiogenesis and stimulating M2-macrophage polarization mediated by the nuclear factor-kappaB (NF-κB) signaling pathway [124]. It was found that ADSC-Exos ameliorated the necrotic area of the flaps, while the overexpression of miR-132 in ADSC-Exos led to further restoration of these flaps. Moreover, the transfection of certain genes can encode genes enhancing exosome production, providing new insights into diabetic wound treatment. As determined by the measurement of luminescent reporter genes, researchers found that cotransfection of parental MSCs with plasmids encoding production-enhancing genes resulted in a significant 15- to 40-fold increase in exosome production [125]. However, this approach encounters several drawbacks, including poor transfection efficiency and unstable gene expression.

In addition to genetic modification, MSC-Exos have also been modified with cargos through prior treatment of the culture surroundings. Compared to exosomes secreted from untreated MSCs, the capacity to facilitate the healing of diabetic wounds can be significantly improved using exosomes derived from pretreated MSCs. Melatonin-pretreated MSC-Exos could accelerate diabetic wound healing by activating the PTEN/AKT signaling pathway, leading to an increase in the M2/M1 polarization ratio, suppression of proinflammatory cytokines such as IL-1β and TNF-α, and enhancement of anti-inflammatory cytokines such as IL-10 [14]. Moreover, melatonin-Exos elevated the proportion of M2-polarized macrophages and enhanced angiogenesis as well as collagen synthesis in the model when compared with exosomes from untreated MSCs. Further studies revealed that drug-pretreated MSC-Exos could affect angiogenesis by the activation of the PI3K/Akt/endothelial nitric oxide synthase (eNOS) signaling pathway via miRNAs. For example, compared with exosomes from nontreated MSCs and control samples, exosomes originating from BMSCs pretreated with atorvastatin promoted diabetic wound repair by enhancing the biological activity of endothelial cells through the AKT/eNOS pathway by upregulating miR-221-3p [126]. Moreover, exosomes derived from hUCMSCs preconditioned with Nocardia rubra cell wall skeleton exhibited superior proangiogenic effects on diabetic wound repair via the circIARS1/miR-4782-5p/VEGFA axis [127]. Compared to untreated exosomes, Nocardia rubra cell wall skeleton-Exos had a robust impact on promoting wound healing *in vivo* by promoting angiogenesis in wounds. Survival and proliferative differentiation of ADSCs were significantly enhanced after hypoxia induction compared to normoxia, and hypoxic ADSC-Exos accelerated the promotion of high-quality healing in diabetic wounds through activation of the PI3K/Akt pathway [35]., It was further observed that the delivery of circ-Snhg11 could improve wound healing in diabetic mice by inducing M2-like macrophage polarization via the miR-144-3p/HIF-1α axis [79]. In addition, compared with control treatment, the upregulation of circ-Snhg11 partially restored endothelial progenitor cells (EPCs) function.

#### Direct MSC-Exos engineering in diabetic wound healing

Since ncRNAs that play an essential role in the development of diabetic wound healing are aberrantly expressed in diabetic wounds, and at the same time are generally less abundant in most natural exosomes [128], researchers have been prompted to endeavor to manipulate the ncRNA content in diabetic wounds by direct MSC-Exos engineering aimed at reinforcing their therapeutic efficacy. In a diabetic wound model, scholars [129] loaded miR-155 inhibitor into exosomes from BMSCs by co-incubation, which promoted the enhancement of keratinocyte migration, the restoration of FGF-7 levels and the exertion of anti-inflammatory effects, and notably improved angiogenesis, collagen deposition and re-epithelialization *in vivo*. The results showed that compared to controls, the miR-155 inhibitor-loaded MSC-Exos achieved maximal wound closure. miR-21 was shown to be involved in cell proliferation and migration and was significantly reduced in diabetic trauma [130]. By introducing miR-21-5p directly into ADSC-Exos via electroporation, the engineered exosomes expedited diabetic wound healing by enhancing the proliferation and migration of keratinocytes through Wnt/β-linker protein signaling, as well as promoting the formation of epithelization, collagen remodeling, revascularization and vessel maturation. Compared to their counterparts, exosomes containing miR-21-5p applied to trauma resulted in elevated expression of CD31 and alpha-smooth muscle actin (α-SMA) [55]. The research further found that a straightforward and groundbreaking technique for equipping peptides has now been developed, which can boost the ability of exosomes to carry various loads (e.g. miRNAs) [131]. A well-known cell-penetrating peptide (YARA) was covalently conjugated with miR-21-5p and subsequently co-incubated with MSC-Exos, thereby significantly augmenting the loading efficiency. Exosomes loaded with YARA-miR-21-5p enhanced the proliferation, migration and invasion of fibroblasts from human and mouse *in vitro* wounds compared to unmodified exosomes and free YARA-miR-21-5p. This technique provides new insight into the use of MSC-Exos containing payloads for diabetic wound therapy.

#### Biomaterial-delivered MSC-Exos in diabetic wound healing

To augment the therapeutic efficacy of exosomes, researchers have combined MSC-Exos with biomaterials, such as hydrogels, to address concerns related to regulating exosome discharge, bacterial infestation, hypoxia and high blood glucose levels in diabetic wounds.

Stimulus-responsive hydrogels react to alterations in the surrounding environment (e.g. pH, enzymes, temperature and light), effectively managing the discharge of MSC-Exos within the intricate environment of diabetic wounds. For example, Wang *et al*. [120] pioneered the development of a long-term pH-responsive, injectable, self-healing and antimicrobial peptide-based FHE hydrogel. The FHE hydrogel was composed of Pluronic F127 (F127), oxidative hyaluronic acid (OHA), and Poly-ε-L-lysine (EPL). The encapsulated ADSC-Exos significantly promoted the proliferation and migration of cells and the tube-forming ability of human umbilical vein endothelial cells (HUVECs) *in vitro* and improved the healing of diabetic all-over skin wounds *in vivo*. Compared with wounds treated with MSC-Exos, wounds treated with FHE@exo hydrogel exhibited more granulation tissue, the shortest wound length and a significant increase in the number of dermal appendages. Conventional PEG hydrogels are incapable of releasing exosomes in response to specific stimuli. Jiang *et al*. [119] loaded ADSC-Exos into MMP-degradable PEG (MMP-PEG) smart hydrogels, which could release exosomes in response to MMP stimulation and subsequently enhance cellular function via Akt signaling while alleviating H_2_O_2_-induced oxidative stress and accelerating diabetic wound healing. The PF-127 hydrogel possesses heat-sensitive properties to accommodate the irregular space of diabetic wounds [132]. The utilization of the PF-127 hydrogel in conjunction with hUCMSC-Exos led to enhanced formation of granulation tissue and significantly faster diabetic wound closure than the use of hUCMSC-Exos alone [109]. In addition, a recent prospective study prepared the required indwelling microneedle, which consisted of MSC-Exos encapsulating adjustable polyvinyl alcohol hydrogel as the tip and 3M removable medical tape as the support matrix. Due to the ion-responsive nature of the hydrogel, released MSC-Exos effectively activated fibroblasts, vascular endothelial cells and macrophages to facilitate diabetic wound healing [113]. The results revealed a faster and most pronounced reduction in the area of diabetic wounds in rats compared with that in rats treated with MSC-Exos. Hence, by incorporating other biomaterial strategies, this new stimuli-responsive hydrogel loaded with MSC-Exos may have practical and clinical potential in diabetic wound healing.

Quite a few studies have also concentrated on refining the properties of hydrogels, including increasing oxygen release and enhancing antimicrobial and tissue adhesion capabilities, to enhance the efficacy of MSC-Exos in diabetic wounds. For example, the OxOBand is a highly porous cryogel with sustained oxygen-release properties. Shiekh *et al*. supplemented OxOBand with ADSC-Exos, which attenuated oxidative stress, increased the migration of human keratinocytes and fibroblasts, promoted the development of mature epithelial structures, and accelerated diabetic wound healing [133]. ADSC-Exos were incorporated into Ag@bovine serum albumin nanoflowers and further encapsulated within injectable collagen hydrogels to simultaneously transform the oxidative wound microenvironment and precisely release the exosomes. Due to the ability of Ag^+^ to activate the wound microenvironment to protect exosomes from oxidative denaturation, this system efficiently eradicated bacteria and induced the apoptosis of damaged oxidized cells, facilitating collagen deposition, neovascularization, angiogenesis and re-epithelialization, and markedly accelerating wound healing in diabetic mice [134]. More importantly, combination therapies involving hydrogels and drug-bioactives have recently been proposed but need to be further explored. Zhang *et al*. [135] designed a novel double-loaded hydrogel with tissue adhesion, antioxidant, self-repair and electrical conductivity properties, and used metformin and ADSC-Exos as therapeutic cargos to reduce ROS levels by interfering with mitochondrial fission, which protected F-actin homeostasis and alleviated microvascular dysfunction to promote chronic diabetic wound healing. Notably, cryogenic 3D printing technology utilizing extrusion was employed to fabricate a 3D scaffolding wound dressing by integrating decellularized small intestinal submucosa, mesoporous bioactive glass and BMSC-Exos. This innovative approach facilitated the sustained release of exosomes, which effectively stimulated the proliferation, migration and angiogenesis of HUVECs, shortened the length of wounds, promoted collagen deposition and accelerated diabetic wound healing compared to exosomes [136].

Hydrogels can also be combined with engineered exosomes to optimize their efficiency in diabetic wound healing. For example, hypoxia-pretreated ADSC-Exos embedded in a GelMA hydrogel delivered circ-Snhg11 to assist in restoring endothelial cell activity to promote diabetic wound healing [137]. They exhibited a more potent therapeutic impact than natural exosomes, as demonstrated by an extended duration of effect and controlled release. The engineering strategy of using reductive 2D covalent organic frameworks (COFs) as nanocarriers to immobilize engineered exosomes from TNF-α-treated MSCs under hypoxia, could be used to suppress oxidative damage and tissue inflammation, promote angiogenesis and eliminate bacterial infections, ultimately leading to a notable enhancement in the healing process of diabetic wounds when exosomes were used alone [138]. For unmodified exosomes, the use of genetically modified exosomes containing miR146a utilizing lentiviral particles, released from a silk fibroin patch, improved the targeting ability and promoted the healing of diabetic wounds by specifically targeting IL-1 receptor-associated kinase 1, reducing inflammation, enhancing granulation tissue formation and neovascularization, and thereby accelerating the healing process of diabetic wounds. As a result, this study not only provided a productive approach to enrich miRNAs in exosomes but also established a pragmatic drug delivery system employing miRNAs to treat diabetic wounds using exosome@biomaterials [69].

### Current status and future prospects

In contrast to acute wounds, MSC-Exos encounter distinct challenges in diabetic wounds including hyperglycemia, adverse effects of advanced glycation end products and a consistently heightened inflammatory microenvironment [139]. Essentially, the main hurdles for MSC-Exos are the presence of abnormal M1 macrophages, impaired vascular maturation, damaged granulation tissue formation and reduced collagen deposition, resulting in delayed healing for diabetic wounds. Bioengineering strategies have been utilized in recent years to increase the production and improve the biological capabilities of engineered MSC-Exos. Currently, in preclinical studies, the application of bioengineered MSC-Exos has been shown to enhance the healing and repair of diabetic wounds.

However, current research on the application of engineered MSC-Exos in diabetic wounds might only be the tip of the iceberg. There are still a multitude of obstacles in the application of engineered MSC-Exos applications. The first obstacle is quality consistency. The absence of standardized techniques and proposals for exosome preparation and separation greatly impacts the pharmacokinetic and pharmacodynamic properties of exosomes, impeding their clinical application. Due to the complexity of biologically active exosome molecules, research in this area is still in its infancy. Further advances in bioengineering technology are required to clarify the underlying targets of exosomes, in order to pave the way for standardized preparation techniques. The second obstacle involves efficacy and safety. Genetic engineering or chemical modification can be employed to enhance Exos-specific cell and tissue targeting. Nevertheless, the impact on the stability of exosomes, the pathway for cell entry and the distribution of exosomes within tissues *in vivo* still requires clarification. Furthermore, more exploration and research are needed to achieve accurate management of the effective loading of cargos into exosomes, extensive manufacturing on a large scale, and minimization of contamination and cytotoxicity. Primarily, combining exosomes with biomaterials requires the consideration of multifunctional hydrogels as extremely versatile scaffold materials. As they exhibit a range of outstanding characteristics, it is inevitable that more complex non-medical components are likely to be added, thus slowing their clinical translation. Therefore, achieving a harmonious equilibrium between efficacy and security might necessitate a thorough assessment.

In future research, exosome engineering technology should focus on developing exosome engineering techniques that produce therapeutic exosomes with increased yield, superb biological activity, efficient drug-carrying capacity and enhanced targeting capabilities. The application of biomaterials with exosomes allows a longer duration of action and better therapeutic effect, and is expected to be implemented in the clinic for actual treatments. Thus, a few promosing eco-friendly biomaterials have become prominent, such as curcumin-containing nanoscaffolds [140] and silver nanoparticles synthesized from olive leaf extracts [141]. In addition, there is also mounting interest in developing 3D printing technologies that allow *in situ* printing for large-scale trauma. Simultaneously, novel medications or molecules can be accommodated into exosomes, enabling targeted delivery to the wound location and achieving effective treatment with minimal drug dosage, which will be of considerable clinical value for treating diabetic wounds. As the most important signaling molecules carried by exosomes, miRNAs profoundly affect cellular functions involved in diabetic wound healing through regulation at the transcriptional and epigenetic levels [142]. As a powerful technology in life science research employed in recent years, single-cell RNA sequencing has dramatically improved our ability to characterize cellular heterogeneity, and when combined with exosomal RNA, it can be used to reconstruct the intricate network of cellular interactions [143], shedding light on the molecular mechanism underlying miRNA therapy for diabetic trauma and leading to the further development of targeted drugs to optimize therapy. The potential future of bioengineered MSC-Exos therapeutics includes combination of targeted exosomes with therapeutic agents and performance-improving biomaterials to construct versatile platforms for *in vivo* tracking, prognostic surveillance and therapeutics.

In summary, experimental preclinical data have confirmed the biocompatibility and safety of bioengineered MSC-Exos for diabetes-related wound treatments [122,129,134]. Additionally, they demonstrate superior biological properties and improved exosome delivery or yield in comparison to natural MSC-Exos, showcasing their capacity to enhance diabetic wound healing in diabetic animal models. However, more exploration is needed to move from preclinical to clinical studies. There are currently 7 published clinical studies and 14 ongoing clinical trials investigating the effectiveness of MSC-Exos in treating various diseases such as acute respiratory distress syndrome, kidney disease, graft vs. host disease, osteoarthritis, stroke, Alzheimer’s disease and type 1 diabetes [144], which signals that the dawn of clinical studies on diabetic wound treatments is coming. Encouragingly, bioengineered MSC-Exos have been used for clinical assessment of the feasibility of drug delivery in cancer therapy. In a groundbreaking clinical advancement, the phase I clinical trial (NCT03608631) will explore the optimal dosage and side effects of MSC-Exos loaded with KrasG12D siRNA for treating pancreatic cancer patients with KrasG12D mutations [145].

Despite the fact that the majority of progressive studies are based on animal models and lack human data, the advancement and refinement of bioengineered MSC-Exos offer a new avenue for precision medicine and can address the shortcomings of natural exosomes-based therapy in diabetic wounds, which appears to be promising in diabetic wound healing. Nevertheless, there is a long way to go from bench to bedside. However, some experiments have already shown us opportunities for future disease treatments and we believe that bioengineered MSC-Exos therapy will become a prominent area of focus.

## Conclusions

MSC-Exos, as carriers for intercellular communication, show great potential for application in diabetic wound therapy. Bioengineering strategies have been leveraged to augment their efficacy and yield. These strategies include parental MSC-Exos engineering, direct MSC-Exos engineering and MSC-Exos-incorporated biomaterials. Characterized by remarkable attributes, bioengineered MSC-Exos have achieved marvelous and desirable results in diabetic wound healing, which signals a positive outlook for the future. Therefore, further exploration of MSC-Exos-based bioengineering strategies will provide new insights into diabetic wound repair.

## Funding

This work was supported by grants from the National Natural Sciences Foundation of China (No. 82160378, 81460293) and Chongqing Traditional Chinese Medicine Inheritance and Innovation Team Project (2023090006KJZX2022WJW008).

## Authors’ contributions

LHL wrote the manuscript. DWL evaluated and reviewed the manuscript. Both authors read and approved the final manuscript.

## Conflict of interest

None declared.
